# Dynamics of Transforming Growth Factor (TGF)-β Superfamily Cytokine Induction During HIV-1 Infection Are Distinct From Other Innate Cytokines

**DOI:** 10.3389/fimmu.2020.596841

**Published:** 2020-11-24

**Authors:** Matthew Dickinson, Anna E. Kliszczak, Eleni Giannoulatou, Dimitra Peppa, Pierre Pellegrino, Ian Williams, Hal Drakesmith, Persephone Borrow

**Affiliations:** ^1^ Nuffield Department of Clinical Medicine, University of Oxford, Oxford, United Kingdom; ^2^ MRC Human Immunology Unit, MRC Weatherall Institute of Molecular Medicine, University of Oxford, Oxford, United Kingdom; ^3^ Computational Genomics Laboratory, Victor Chang Cardiac Research Institute, Sydney, NSW, Australia; ^4^ St Vincent’s Clinical School, University of New South Wales, Sydney, NSW, Australia; ^5^ Mortimer Market Centre, Department of HIV, CNWL NHS Trust, London, United Kingdom; ^6^ Centre for Sexual Health and HIV Research, University College London, London, United Kingdom

**Keywords:** HIV, TGF-beta, activin, interferon, cytokine, dendritic cell, innate response, bone morphogenetic protein

## Abstract

Human immunodeficiency virus type 1 (HIV-1) infection triggers rapid induction of multiple innate cytokines including type I interferons, which play important roles in viral control and disease pathogenesis. The transforming growth factor (TGF)-β superfamily is a pleiotropic innate cytokine family, some members of which (activins and bone morphogenetic proteins (BMPs)) were recently demonstrated to exert antiviral activity against Zika and hepatitis B and C viruses but are poorly studied in HIV-1 infection. Here, we show that TGF-β_1_ is systemically induced with very rapid kinetics (as early as 1–4 days after viremic spread begins) in acute HIV-1 infection, likely due to release from platelets, and remains upregulated throughout infection. Contrastingly, no substantial systemic upregulation of activins A and B or BMP-2 was observed during acute infection, although plasma activin levels trended to be elevated during chronic infection. HIV-1 triggered production of type I interferons but not TGF-β superfamily cytokines from plasmacytoid dendritic cells (DCs) *in vitro*, putatively explaining their differing *in vivo* induction; whilst lipopolysaccharide (but not HIV-1) elicited activin A production from myeloid DCs. These findings underscore the need for better definition of the protective and pathogenic capacity of TGF-β superfamily cytokines, to enable appropriate modulation for therapeutic purposes.

## Introduction

Effective prophylactic strategies are urgently required to halt the continued spread of the human immunodeficiency type 1 (HIV-1) pandemic. Furthermore, although an increasing proportion of HIV-infected individuals are now accessing combination antiretroviral therapy (ART) (1), there is a need for improved therapeutic regimes, both to facilitate long-term control (or even achieve a functional cure) of established infection and to block the ongoing inflammation and immune activation that accompanies even well-controlled HIV-1 replication, which is associated with substantial long-term morbidity (2). The rational design of novel strategies to reduce HIV-1 transmission and ameliorate the consequences of established infection has been revolutionized by recent advances in understanding of the immune responses activated following HIV-1 infection and their roles in protection and/or pathogenesis (3), and there is an important need for further insight into HIV-immune system interactions at different stages of infection to advance this field.

Disseminated viral replication in acute HIV-1 infection induces a systemic cytokine storm, with elevations in circulating levels of multiple cytokines and chemokines including type I interferons (IFNs) occurring as viremia escalates (4–6). Although the high-magnitude analyte elevations triggered prior to peak viremia are not sustained as viral replication declines, low-level perturbations continue into chronic infection (7, 8), reflecting dysregulated inflammation and immune activation (9). Analyte levels also fail to normalize after initiation of antiretroviral therapy (ART) (7, 10) as chronic inflammation persists (11). Innate cytokines such as type I IFNs mediate both beneficial and detrimental effects during HIV-1 infection, inhibiting viral replication (12, 13) but contributing to immune activation and pathogenesis (14). The lack of pathogenesis of simian immunodeficiency virus (SIV) infections in their natural hosts is associated with rapid down-regulation of IFN responses and systemic inflammation after acute infection (15, 16).

The transforming growth factor (TGF)-β superfamily is a group of pleiotropic cytokines that includes the three isoforms of TGF-β (TGF-β_1-3_), three activins (A, B and AB), bone morphogenetic proteins (BMPs) and others (17). Like IFNs, TGF-β superfamily members have pleiotropic effects on the activation, proliferation and differentiation of multiple cell types, including immune cell subsets (18–20). Recent research has also revealed that BMPs and activins exhibit antiviral activity against diverse RNA and DNA viruses including hepatitis B virus (HBV), hepatitis C virus (HCV) and Zika virus (21, 22) through induction of an IFN-like transcriptional signature, with BMPs both enhancing type I IFN-mediated antiviral activity and also inhibiting viral replication independently of IFN (22). Activin A has also been shown to prevent reactivation of human cytomegalovirus (HCMV) in dendritic cells (DCs) *in vitro* (23).

Prior studies in non-human primate infection models have shown that a marked increase in plasma TGF-β_1_ levels is induced within 24 h of intravenous inoculation of African green monkeys with SIVagm (a non-pathogenic infection model), but that this rapidly resolves (24). By contrast, in the pathogenic rhesus macaque SIVmac infection model, the initial increase in circulating TGF-β_1_ levels is more modest, and plasma TGF-β_1_ levels remain elevated throughout acute and early infection (24). Likewise, although the kinetics of TGF-β_1_ induction during the earliest stages of HIV-1 infection have not been characterized, plasma TGF-β_1_ levels in HIV-infected individuals studied during subacute and chronic infection have been found to be higher than those in HIV-seronegative control subjects (25–27), and correlate positively with levels of T cell activation in early infection (25) and with greater CD4^+^ T cell loss and enhanced disease progression during chronic infection (26, 27), However, with the exception of TGF-β_1_, the roles of which in HIV-1 infection are complex and as yet incompletely understood (28), the TGF-β superfamily remains poorly studied in HIV-1 infection.

To gain insight into the extent and kinetics of systemic induction of different TGF-β superfamily cytokines during natural HIV-1 infection, this study sought to measure levels of TGF-β_1_, activins A and B and BMP-2 in plasma samples from HIV-infected individuals during the acute, subacute and chronic phases of HIV-1 infection. Systemic induction of TGF-β superfamily members was found to differ markedly from that of many other innate cytokines (including IFN-α), with a modest but extremely rapid increase in plasma TGF-β_1_ concentrations occurring as viremia first began to increase in acute HIV-1 infection that was then sustained throughout acute infection and into chronicity, whilst plasma concentrations of BMP-2 and activins A and B were largely unperturbed. Whereas *in vitro* exposure of DC subsets to HIV-1 elicited high levels of IFN-α production from plasmacytoid (p)DCs, it failed to trigger production of TGF-β_1_, BMP-2 or activins A and B from either pDCs or myeloid (m)DCs, suggesting the importance of differences in the principal cellular sources of production of TGF-β superfamily and other innate cytokines in determining their differing *in vivo* induction patterns.

## Materials and Methods

### Human Samples

Longitudinal plasma samples from US plasma donors who initially showed no evidence of HIV-1 infection but during a serial plasma donation timespan were retrospectively found to have undergone HIV-1 seroconversion (4) were obtained from Zeptometrix Corporation (USA). Samples had been collected by plasmapheresis (a process that separates plasma from all the cellular components present in blood, including platelets) into 4% sodium citrate and cryopreserved. Serial samples from each donor cryopreserved at time-points prior to systemic detection of viremia (as determined by Quest Diagnostics Incorporated (USA) with a Roche Amplicor HIV Ultra assay (lowest level of detection 100 viral copies/ml)) and during the phase of exponentially increasing viremia in acute infection were selected for analysis.

Whole blood from individuals recruited following clinical presentation with HIV-1 infection at the Mortimer Market Sexual Health Clinic (London, UK) and sampled during subacute and/or chronic infection was drawn into EDTA collection tubes, and plasma was separated and cryopreserved. Blood samples were processed within 6 h of withdrawal by diluting in 0.9% saline, underlaid with Histopaque-1077 (Sigma-Aldrich, USA), mononuclear cells separated by removal of the buffy coat, and erythrocytes/platelets pelleted by centrifugation. Diluted plasma was aliquoted and frozen at −80°C for later use. Demographically matched seronegative control subjects were also recruited at the Mortimer Market Sexual Health Clinic, and plasma separated from blood samples was similarly cryopreserved. The vast majority of individuals were men who have sex with men (MSM) and of Caucasian ethnicity.

PBMCs were isolated by Histopaque (Sigma-Aldrich, USA) density gradient centrifugation from leukocyte reduction system chamber samples from healthy platelet donors that were purchased from NHS Blood & Transplant, UK. Assays involving analysis of pDC function were performed on fresh, not cryopreserved samples.

### Viruses

HIV-1 NL4-3 was generated from an infectious molecular clone (IMC) originally obtained from Drs John Kappes and Christina Ochsenbauer (UAB, USA). 10^7^ 293 T cells in 75 cm^2^ surface area flasks were transfected with HIV-1 DNA using Lipofectamine 2000 (Sigma-Aldrich, USA). 12 μg HIV-1 DNA in 1 ml Opti-MEM media (Thermo Fisher Scientific, USA) was combined with 25 μl Lipofectamine 2000 also in 1 ml Opti-MEM. After allowing Lipofectamine-DNA complexes to form for 30 min at room temperature, the mixture was added to 8 ml of DMEM (Thermo Fisher Scientific, USA) that had been supplemented with 10% FBS, 2 mM l-glutamine, 1% non-essential amino acids and 100 IU/ml penicillin plus 100 μg/ml streptomycin and added to the flask. Virus-containing supernatants were harvested after 72 h. Reverse transcriptase activity was determined using a colorimetric reverse transcriptase kit (Roche Diagnostics, Switzerland). The PR8 influenza virus stock (which had been produced and titered in MDCK-SIAT1 cells (29, 30)) was a kind gift from Alain Townsend, University of Oxford, UK.

### ELISA and Luminex® Assays

Activin A was measured using a human/mouse/rat activin A Quantikine® ELISA kit (Bio-Techne, USA). Activin B was measured by ELISA (Cloud-Clone Corp, USA). BMP-2 was measured using a BMP-2 Quantikine® ELISA kit (Bio-Techne, USA). IL-18 was measured using a human total IL-18 Quantikine® ELISA kit (Bio-Techne, USA). IFN-α was measured using a VeriKine human interferon alpha ELISA kit (PBL Assay Science, USA). PF4 was measured using a human PF4 Quantikine® ELISA kit (Bio-Techne, USA). Total (active and latent) TGF-β_1_ was measured in plasma using a TGF-β_1_ Magnetic Luminex® Performance Assay kit (Bio-Techne, USA) and in cell culture supernatants using a TGF-β_1_ Quantikine® ELISA kit (Bio-Techne, USA) that both include an acid activation step prior to the assays. The manufacturer reports no significant cross-reactivity with other TGF-β isoforms interfering with the measurement of TGF-β_1_ in these assays. Lipopolysaccharide (LPS) was measured with a Kinetic-QCL™ LAL assay (Lonza, Switzerland). All assays were run according to the manufacturer’s instructions, in technical duplicate or triplicate. The coefficient of variation (CV%) was measured for all replicates, and only readings with an intra-well CV% of <20% were accepted.

As plasma levels of certain cytokines have been shown to be affected by long term sample cryostorage and freeze-thawing (31–33) or anticoagulants (31, 32, 34), quality control experiments were initially performed to address whether freeze-thawing or anticoagulant choice impacted on levels of the analytes measured here. Neither freeze-thawing nor anticoagulant use (EDTA, lithium heparin or 4% sodium citrate) was found to have a significant effect on plasma concentrations of TGF-β_1_, activins A and B or BMP-2 (**Supplementary Figure 1**).

### Dendritic Cell Isolation and Culture

Primary CD1c^+^/CD141^+^ mDCs were isolated from PBMCs by negative selection using a MACS® myeloid dendritic cell isolation kit (Miltenyi Biotec, Germany). pDCs were isolated from PBMCs by negative selection using a MACS® plasmacytoid dendritic cell isolation kit II (Miltenyi Biotec, Germany). Cells were cultured in R10 medium consisting of RPMI-1640 medium (Life Technologies, USA) supplemented with 10% FBS (Sigma-Aldrich, USA), 2 mM l-glutamine (GlutaMAX, Life Technologies, USA) and 100 units/ml penicillin plus 100 μg/ml streptomycin (Life Technologies, USA).

### *In Vitro* Analysis of Cytokine Production by DC Subsets

0.3–1.5 × 10^5^ pDCs or 0.3–0.7 × 10^5^ mDCs were plated/well into duplicate wells in flat-bottomed 96-well plates (Corning Inc, USA) and cultured in a total volume of 200 μl/well R10 in the presence or absence of HIV-1 NL4-3 (50 ng RT/ml), PR8 influenza virus (MOI 10, based on plaque-forming units (PFU)/ml), LPS (100 ng/ml) (Sigma-Aldrich, USA), R837 (5 μg/ml) (Invivogen, USA), R848 (10 μg/ml) (Invivogen) or ODN 2216 (5 μM) (Invivogen). After incubation for 24 h at 37°C in 5% CO_2_ supernatants were harvested and cytokine concentrations were analyzed by ELISA or Luminex® assay. Values for duplicate test wells were averaged.

### Statistics

**Figure 1:**
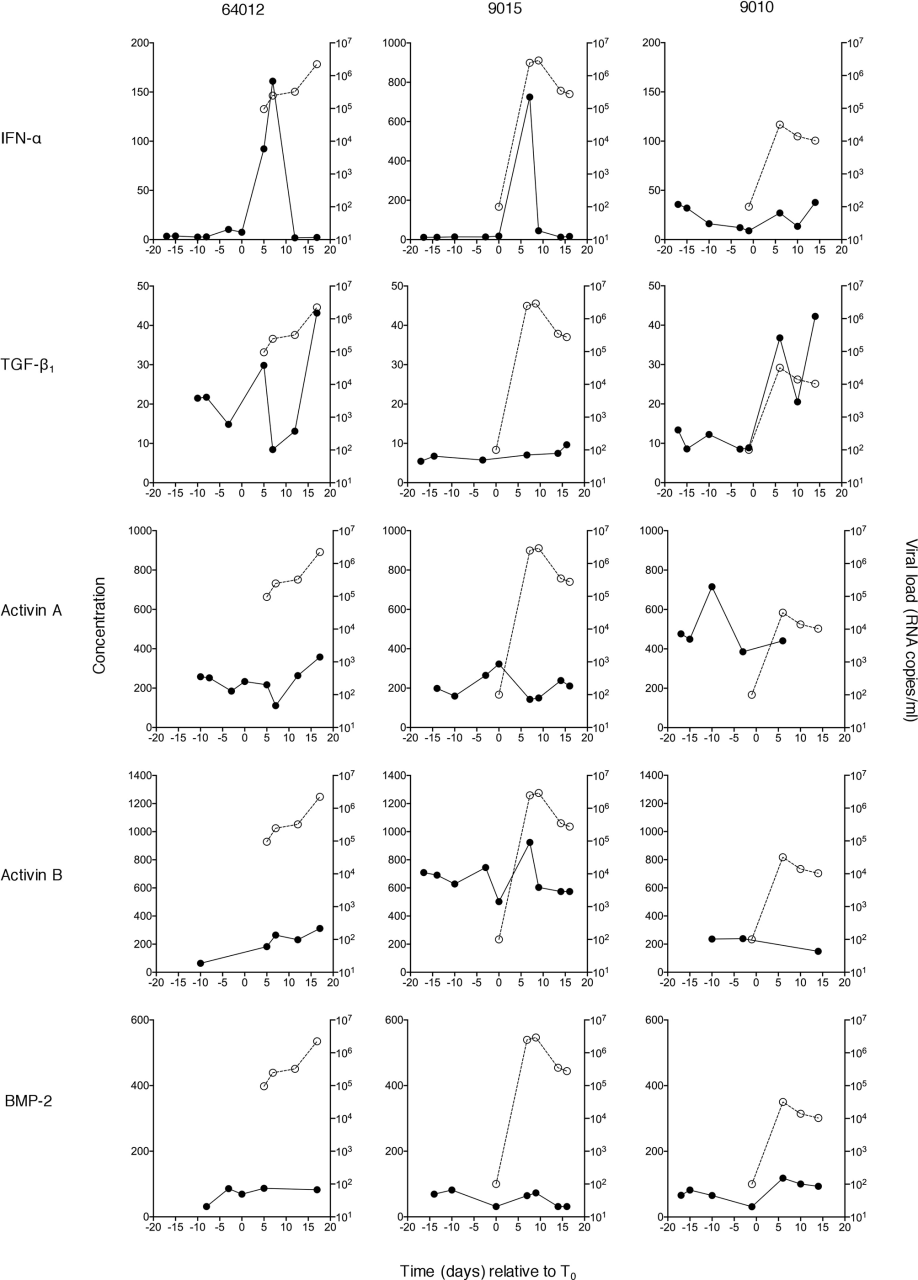
Plasma concentrations of IFN-α and TGF-β superfamily cytokines at serial time-points during acute HIV-1 infection in three donors. Circulating concentrations of TGF-β_1_, activin A, activin B and BMP-2 were measured in serial plasma samples collected at time-points both pre- and post-T_0_ (the time-point when viral load first reached detectable levels (100 RNA copies/ml)) from plasma donors who became acutely infected with HIV-1. Data from three representative subjects is shown. Plasma IFN-α levels in the same individuals (evaluated as part of a prior study (4) are shown for comparison. Closed circles/solid lines represent cytokine concentrations (pg/ml, except TGF-β_1_ which is in ng/ml); open circles/dashed lines represent plasma viral load (RNA copies/ml).

**Figure 2:**
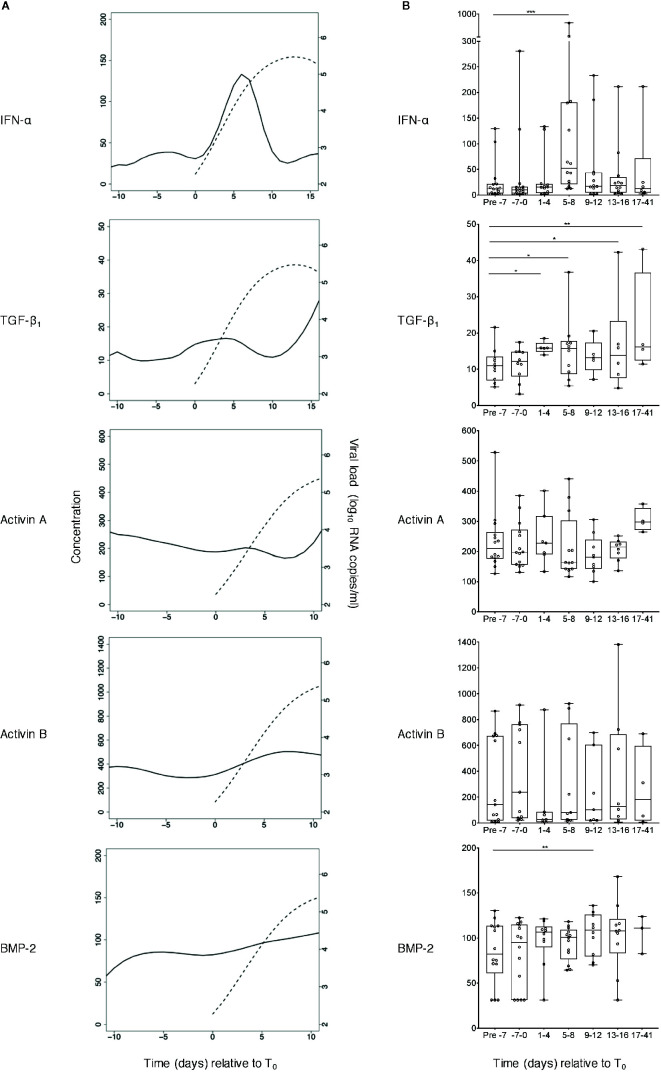
Perturbations in plasma concentrations of IFN-α and TGF-β superfamily cytokines during acute HIV-1 infection. **(A)** TGF-β_1_, activin A, activin B and BMP-2 concentrations were measured in plasma samples collected at time-points both pre- and post-T_0_ (the time-point when viral load first reached detectable levels (100 RNA copies/ml)) from plasma donors who became acutely infected with HIV-1 during the time-frame of serial plasma donation. IFN-α levels in the same samples had already been evaluated as part of a prior study (5). Data from n=15 (IFN-α), n=10 (TGF-β_1_), n=14 (activin A), n=13 (activin B), or n=14 (BMP-2) donor time-courses were combined and plotted using cubic interpolation splines. Solid lines represent cytokine concentrations (pg/ml, except TGF-β_1_ which is in ng/ml), and dashed lines represent viral load (RNA copies/ml). **(B)** Pooled data were binned, and the pre- day −7 (relative to T_0_) bin was used as a baseline to which other bins were compared, using linear mixed-effects modelling (*P < 0.05, **P < 0.01, ***P < 0.001). Box plots represent the limits of the 25th and 75th percentile, with the central line showing the median. Whiskers represent the range. All cytokine concentrations are in pg/ml, except TGF-β_1_ which is in ng/ml.

To enable longitudinal analysis of alterations in systemic cytokine concentrations during acute HIV-1 infection in the US plasma donor cohort (**Figures 1** and **2**), data from the different donors was temporally aligned based on T_0_ (the time-point when viremia first reached 100 RNA copies/ml plasma that was estimated using linear mixed-effects (LME) modelling as previously described (4)). Interpolation curves were created from longitudinal plasma cytokine data using the R package *fields* as previously described (35). LME models were used to compare cytokine concentrations of binned pre-day −7 samples to those in post-day −7 bins using R packages *nlme* and *lme4* as previously described (35).

**Figure 3:**
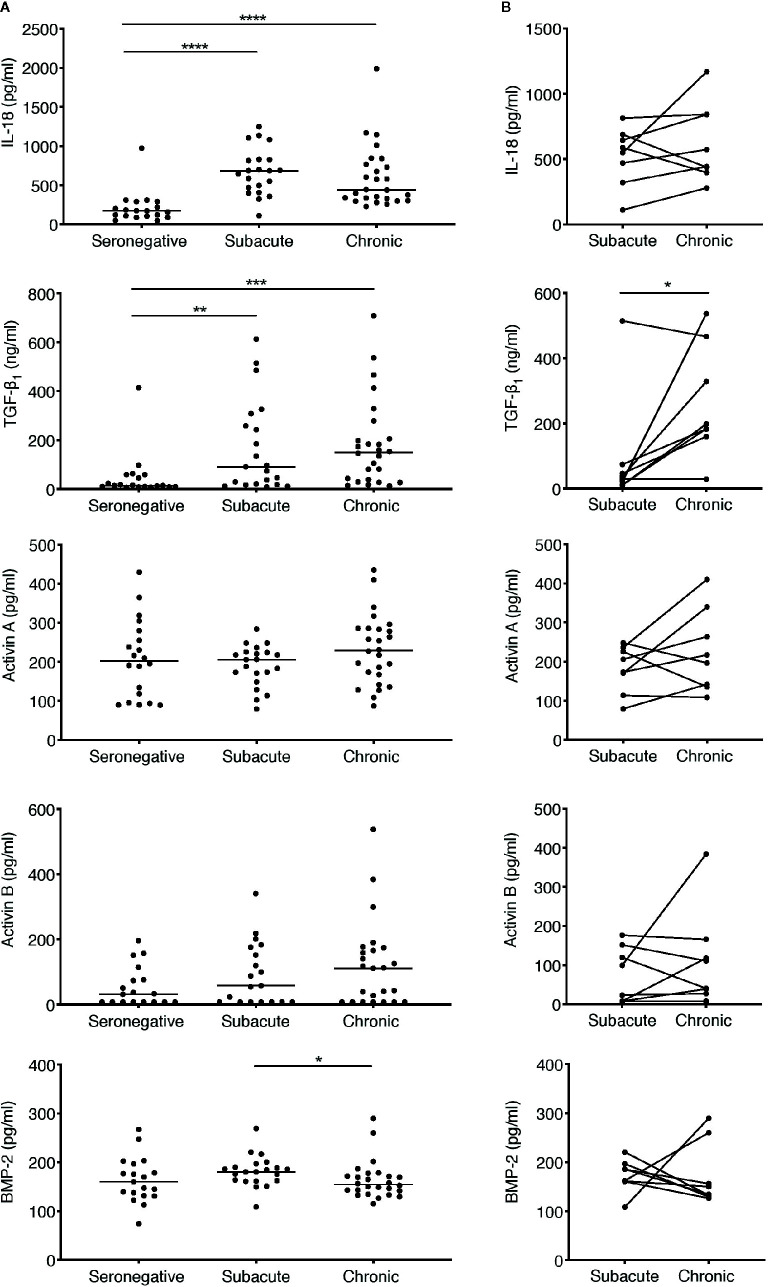
Analysis of perturbations in plasma concentrations of IL-18 and TGF-β superfamily cytokines in subacute and chronic HIV-1 infection. **(A)** Concentrations of TGF-β superfamily cytokines and IL-18 were measured in plasma samples from HIV-infected individuals sampled cross-sectionally during subacute (within 30 days FOSx) or chronic (> 2 years FOSx) untreated infection, and in demographically matched HIV-seronegative control subjects. Symbols represent datapoints from individual subjects (n of datapoints included from seronegative, subacute and chronic groups: IL-18 and TGF-β_1_ n=19, 21, 26; activin A n=20, 21, 26; activin B n=18, 19, 25; BMP-2 n=19, 21, 26); and horizontal lines denote group medians. The statistical significance of differences between groups was determined by Kruskal-Wallis test with Dunn’s post-test. **(B)** Samples were available from n=8 patients at time-points in both subacute and chronic untreated infection. Inter-time-point differences in cytokine levels in these subjects were compared by Wilcoxon signed-rank test (*P < 0.05, **P < 0.01, ***P < 0.001, ****P < 0.0001).

**Figure 4:**
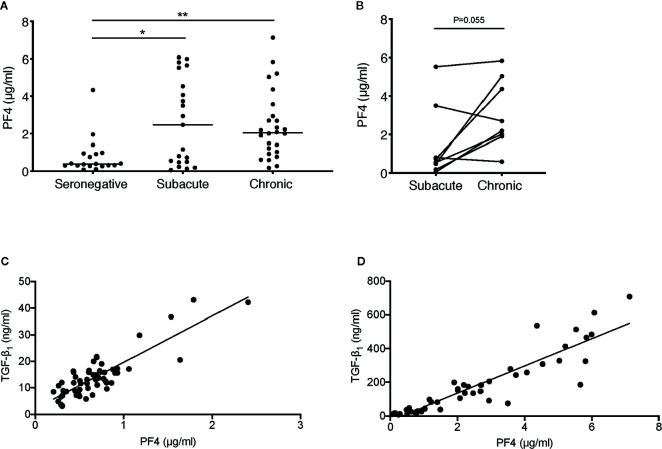
Correlation between plasma concentrations of PF4 and TGF-β_1_ in HIV-1−infected individuals. **(A)** PF4 levels were measured in plasma samples from the HIV-infected individuals sampled cross-sectionally during subacute (within 30 days FOSx) or chronic (> 2 years FOSx) untreated infection and demographically matched HIV-seronegative control subjects studied in **Figure 3**. Symbols represent datapoints from individual subjects (datapoints included: n=19 seronegative, n=21 subacute and n=26 chronic); and horizontal lines denote the group median. The statistical significance of differences between groups was assessed by Kruskal-Wallis test with Dunn’s post-test (*P < 0.05, **P < 0.01). **(B)** Paired data from n=8 subjects from whom samples were available at time-points in both subacute and chronic infection were analyzed by two-tailed Wilcoxon signed-rank test. **(C)** Correlation between TGF-β_1_ and PF4 concentrations in n=56 samples from n=9 plasma donors acutely infected with HIV-1, analyzed by two-tailed Spearman’s rank correlation coefficient (r=0.71, P<0.0001). **(D)** Correlation between TGF-β_1_ and PF4 concentrations in n=47 samples from n=39 individuals sampled during subacute and/or chronic HIV-1 infection, analyzed by two-tailed Spearman’s rank correlation coefficient (r=0.94, P < 0.0001).

**Figure 5:**
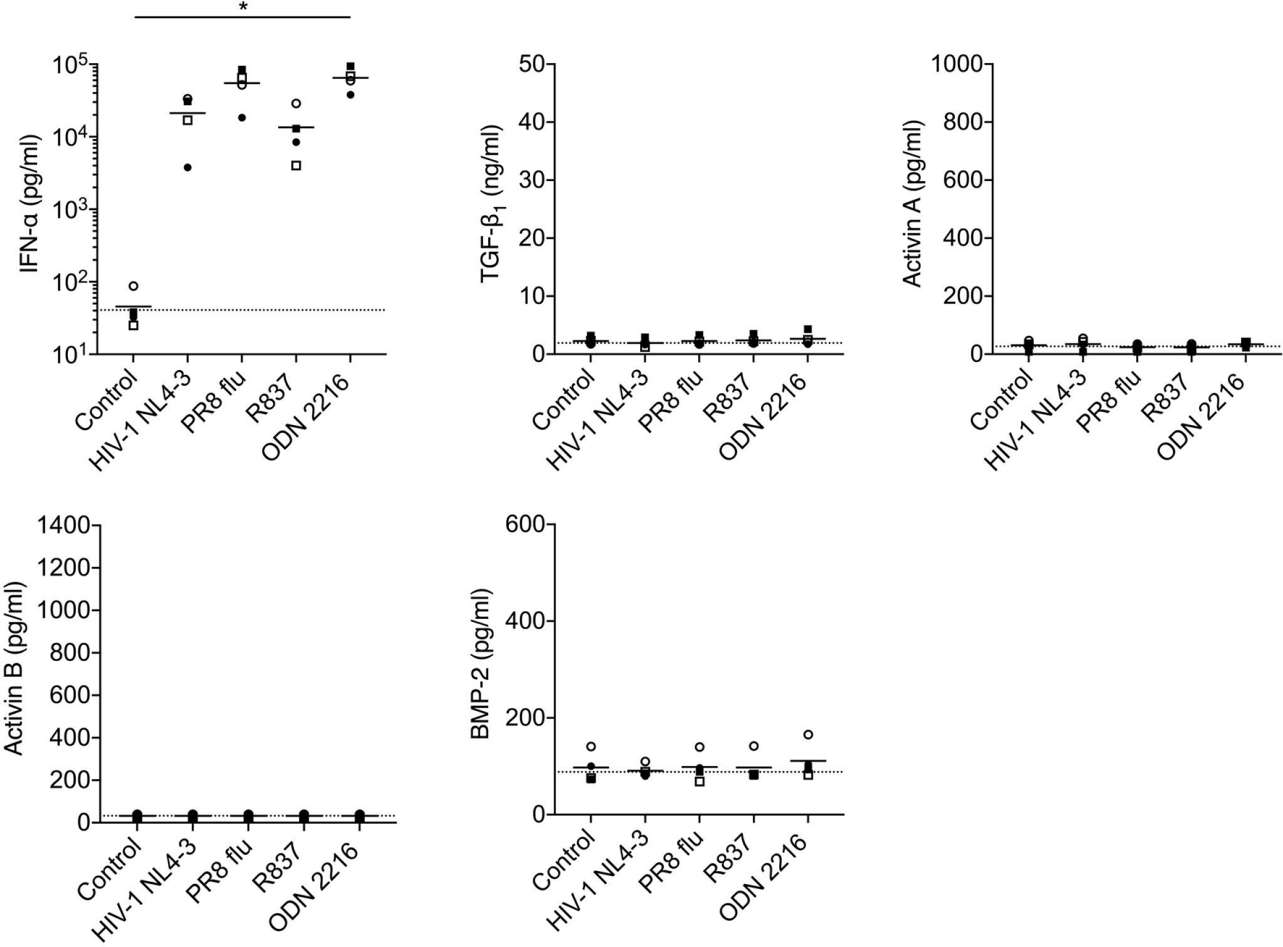
Analysis of production of TGF-β superfamily cytokines by pDCs following *in vitro* stimulation with HIV-1 or TLR ligands. pDCs isolated from n=4 HIV-1-seronegative donors were cultured with medium only, HIV-1 NL4-3, PR8 influenza virus, the TLR7 agonist R837, or the TLR9 agonist ODN 2216. Concentrations of IFN-α and TGF-β superfamily cytokines in supernatants were measured after 24 h. Symbols indicate datapoints from individual donors; horizontal solid lines represent group means. The horizontal dashed line shows background analyte levels in serum-containing medium (mean of values measured in two independent experiments). Data were analyzed using a one-way ANOVA with Dunnett’s post-test comparing to the unstimulated pDC control (*P < 0.05).

**Figure 6:**
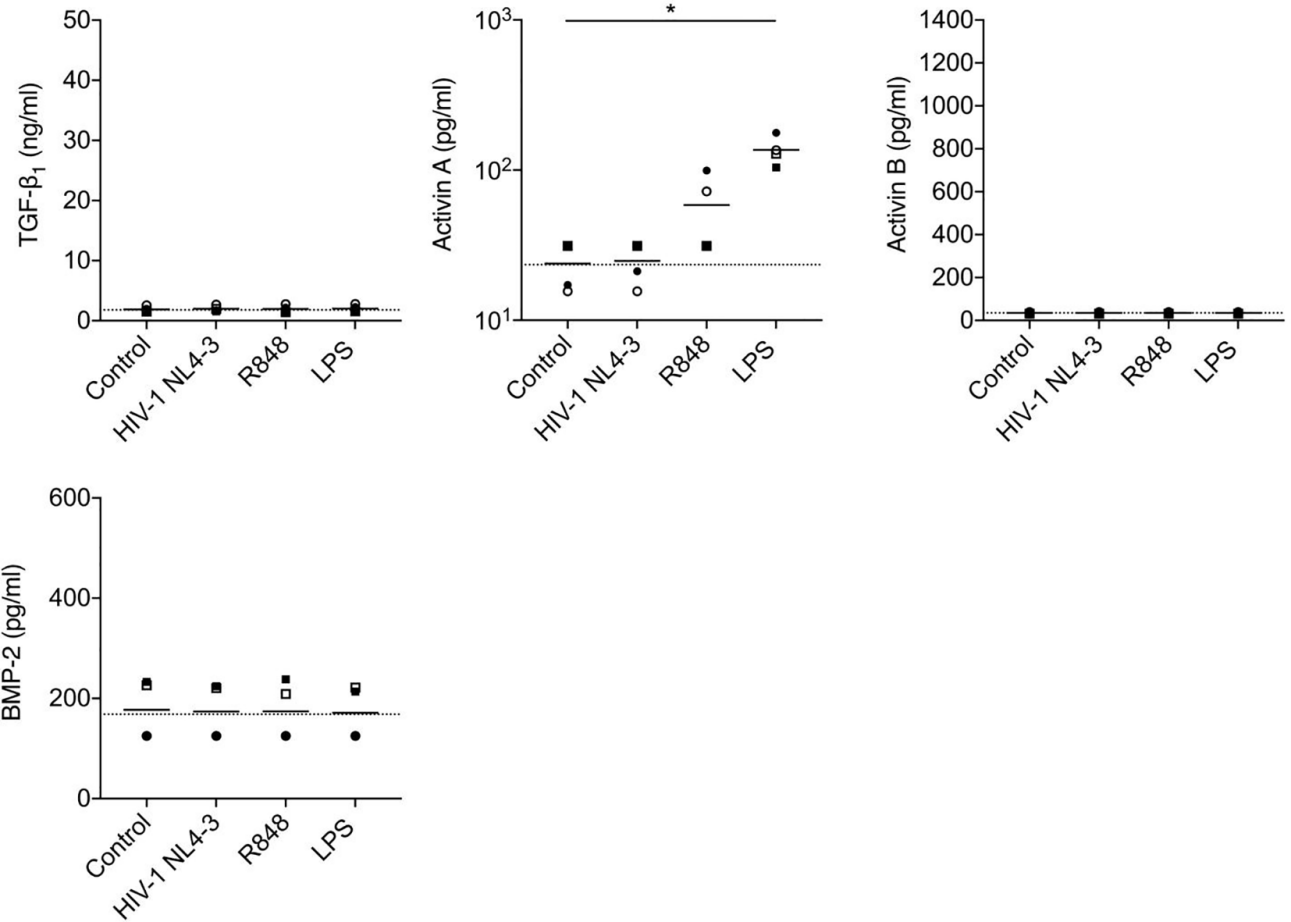
Analysis of production of TGF-β superfamily cytokines by mDCs following *in vitro* stimulation with HIV-1 or TLR ligands. mDCs from n=4 healthy donors were cultured with medium only, HIV-1 NL4-3, TLR7/8 agonist R848 or LPS. Concentrations of TGF-β superfamily cytokines in supernatants were measured after 24 h. The horizontal dashed line shows background analyte levels in serum-containing medium (mean of values measured in two independent experiments). Symbols represent data from four individual donors, solid lines indicate the group means. Data were analyzed using a one-way ANOVA with Dunnett’s post-test comparing each group to the unstimulated mDC control (*P < 0.05).

Statistical analysis of other data was performed using GraphPad Prism version 8 (GraphPad Software Inc, USA). For cross-sectional data involving more than 2 groups (**Figures 3A** and **4A**), groups were compared by Kruskal-Wallis test with Dunn’s post-hoc test for multiple comparisons. For paired analysis of cross-sectional data (**Figures 3B** and **4B**), two groups were compared by two-tailed Wilcoxon signed-rank test. Correlations were analyzed using two-tailed Spearman’s rank correlation coefficient (**Figures 4C–D**). *In vitro* experiments analyzing induction of TGF-β superfamily cytokines by DC subsets (**Figures 5** and **6**) were analyzed using a one-way ANOVA with Dunnett’s (“many-to-one”) post-test, comparing all groups to the medium control.

## Results

### Circulating Levels of TGF-β_1,_ But Not Those of BMP-2 Or Activins A and B, Are Elevated in Acute HIV-1 Infection

To elucidate the systemic kinetics of TGF-β superfamily cytokines during acute HIV-1 infection, serial samples were studied from regular plasma donors who had become infected with HIV-1 during the plasma donation time-course, which provided a unique opportunity to gain insight into the systemic changes in analyte levels during the very earliest stages of HIV-1 infection (4). No information was available about donor identity or demographics. Plasma HIV-1 RNA levels in these samples had already been analyzed (4). The time-points selected for study included baseline samples taken a maximum of 132 days before viremia reached detectable levels (100 copies/ml viral RNA, referred to as the “time origin” or T_0_), and subsequent serial samples, typically drawn at 2- to 5-day intervals for up to 41 days post T_0_.

TGF-β_1_, activin A and B and BMP-2 levels were measured in serial plasma samples from each donor. Examples of viral load and cytokine data from three representative subjects are shown in **Figure 1**; IFN-α concentrations in the same samples, measured as part of a prior study (4) have also been plotted to allow comparison with an innate antiviral cytokine known to be systemically upregulated as viremia increases during acute HIV-1 infection. As anticipated, some inter-donor variability in baseline analyte levels and minor temporal fluctuation in intra-donor analyte levels was observed; and a transient elevation in IFN-α was apparent in two of these subjects. Two of the three donors showed evidence of an increase in plasma TGF-β_1_ levels during the HIV-1 expansion phase, but there was no convincing evidence of upregulation of activin A, activin B or BMP-2 following HIV-1 acquisition. To enable robust quantitative interrogation of perturbations in analyte levels over time, cytokine concentration and viral load data from 10 to 15 donors (depending on the analyte) were pooled and plotted as smoothed curves using spline interpolation (**Figure 2A**). Data were grouped into bins relative to T_0_, and linear mixed-effects models were fitted on the data, which take into account the variability between samples (**Figure 2B**). As perturbations in systemic levels of some analytes have been observed as early as 7 days before T_0_ (36), the mean plasma concentration of each analyte at time-points prior to day −7 was used as a baseline to which the mean values of other bins were compared to enable determination of alterations in cytokine concentrations during acute HIV-1 infection. Plasma levels of TGF-β_1_ were significantly elevated as early 1 to 4 days post-T_0_ and remained similarly upregulated throughout acute infection (**Figure 2B**). By contrast, concentrations of activins A and B remained largely unchanged over the course of acute HIV-1 infection (**Figure 2B**). There was a mild elevation in systemic BMP-2 concentration in acute HIV-1 infection at 9 to 12 days post-T_0_ (**Figure 2B**). However, this particular time-point included fewer donors than the baseline bin, which may have skewed the data.

### Systemic Elevation of TGF-β_1_, But Not BMP-2 or Activins A and B, Is Observed in Subacute and Chronic HIV-1 Infection

To address whether TGF-β superfamily cytokines are perturbed at subsequent stages of HIV-1 infection, plasma samples were obtained from a distinct cohort of subjects recruited following clinical presentation with acute HIV-1 infection. The majority of patients in this cohort were Caucasian MSM infected with HIV-1 subtype B. Plasma samples were available from n=21 subjects at a time-point during subacute infection, which was defined as the timeframe up to 30 days following the onset of acute retroviral syndrome symptoms (FOSx) (sampling time-points ranged from 5 to 29 days FOSx, median 12 days). All subacute infection samples were obtained prior to commencement of ART. A subset of the patients in this cohort declined ART and plasma samples were available from n=26 individuals (including 8 of the 21 subjects from whom samples were also available during subacute infection) at a chronic infection time-point more than two years FOSx, prior to which they had not received ART (range, 753–1,570 days, median 1,243 days). The time-points at which infected individuals were studied, together with plasma viral load and CD4 count data, are shown in **Table 1**. Plasma samples from n=20 demographically matched HIV-seronegative subjects were studied in parallel, to enable cross-sectional analysis of plasma cytokine levels in control subjects, during subacute HIV-1 infection and during chronic untreated HIV-1 infection. Notably, the fact that 8 of the patients in the subacute infection group declined ART and were also sampled during chronic untreated infection enabled analysis of paired data from both time-points in this subset of individuals. Circulating levels of IFN-α in most samples were below the limit of assay detection (data not shown), consistent with prior studies, which have shown that IFN-α is only transiently elevated prior to the peak in viremia in acute infection (4) and is commonly below detectable levels in chronic infection (37, 38). To provide comparative data for an innate cytokine expected to be detectably elevated in the circulation of viremic individuals, we assayed plasma levels of IL-18, a cytokine that has previously been shown to be strongly upregulated during acute infection (4) and then to decline in concentration but nonetheless remain slightly elevated during subacute and chronic infection (37, 38). Cross-sectional comparison of cytokine levels in seronegative controls and subjects sampled during subacute or chronic infection is shown in **Figure 3A**. Paired data from n=8 donors included in both the subacute and chronic infection groups is shown in **Figure 3B**. Consistent with observations made in other cohorts (37, 39), circulating IL-18 levels in the subjects studied here were found to be significantly higher than those in the seronegative control group during both subacute and chronic infection (**Figure 3A**). Plasma TGF-β_1_ levels were likewise found to be significantly higher in both subacute and chronic infection than those in seronegative controls (**Figure 3A**), and there was a statistically significant increase in circulating TGF-β_1_ levels from subacute to chronic infection (**Figure 3B**). By contrast, plasma concentrations of activins A and B and BMP-2 did not differ significantly from those in the seronegative controls (**Figure 3A**), although there was a trend for higher circulating levels of activins A and B in the chronic compared to the subacute infection group (**Figure 3B**). In order to assess whether microbial translocation could have driven this trend, LPS levels were measured in a subset of chronic donors from which enough plasma was remaining. No significant correlation was observed between the concentrations of LPS and activin A in these samples (data not shown).

**Table 1:** Clinical data from donors subacutely and chronically infected with HIV-1.

Group	Subject ID	Days following onset of symptoms	Plasma viral load (RNA copies/ml)	CD4 count (cells/μl)	Group	Subject ID	Days following onset of symptoms	Plasma viral load (RNA copies/ml)	CD4 count (cells/μl)
Subacute	MM12	16	1,555,700	500	Chronic	MM1	1370	29,700	1020
	MM19	13	5,678,900	400		MM4	1222	233,400	490
	MM20	16	2,253,700	220		MM8	801	105,200	240
	MM22	12	9,059,000	ND**^b^**		MM9	945	217,600	160
	**MM23^a^**	9	11,105,300	330		MM12	900	304,800	200
	**MM24**	16	157,700	580		MM13	1125	8,800	240
	**MM28**	6	4,337,100	560		MM14	1394	19,700	340
	**MM31**	20	324,000	370		**MM23**	1064	45,700	240
	MM32	10	147,640,000	320		**MM24**	1322	ND	220
	**MM33**	12	1,451,400	940		MM27	1329	336,300	310
	**MM36**	6	38,417,000	320		**MM28**	1381	15,900	290
	MM38	29	2,721,100	410		**MM31**	1220	153,000	750
	**MM39**	5	350,600	610		**MM33**	1441	30,000	440
	MM41	14	3,172,500	190		MM34	1396	14,000	490
	**MM65**	10	5,000,000	500		**MM36**	1395	216,500	ND
	MM67	24	8,300,000	90		**MM39**	1206	280,000	190
	MM69	12	5,100,000	480		MM40	1530	310,000	250
	MM70	18	2,500,000	260		MM45	1570	1,200	410
	MM72	5	2,100,000	510		MM46	938	160,000	240
	MM73	11	3,700,000	510		MM47	1263	24,000	210
	MM75	9	30,000,000	320		MM51	906	27,000	560
						MM55	1299	11,000	490
						MM56	802	300	620
						MM57	971	130,000	350
						MM62	1173	20,000	1200
						**MM65**	753	18,000	880

^a^Donors sampled in both subacute and chronic infection are indicated in bold.

^b^Not determined.

### Elevated TGF-β_1_ Correlates with Increased Platelet Degranulation in HIV-1 Infection

Platelets are a major source of TGF-β_1_ and release this cytokine upon activation and degranulation, along with alpha-granule chemokines such as CXCL4, also known as platelet factor 4 (PF4) (40). To assess platelet activation in subacute and chronic HIV-1 infection, cross-sectional samples from the subjects studied in **Figure 3** were analyzed for PF4. Plasma PF4 was found to be significantly elevated in both subacute and chronic HIV-1 infection (**Figure 4A**) indicating marked platelet activation. In n=8 donors from whom paired samples in subacute and chronic infection were available, there was no statistically significant increase in circulating PF4 concentration between these time-points (**Figure 4B**).

PF4 was also measured in the samples from the acutely infected US plasma donor samples studied in **Figure 2**, where it was found to be positively correlated with TGF-β_1_ concentration (r=0.71, P<0.0001) (**Figure 4C**). Similarly, when considering subacutely and chronically HIV-infected subjects together, PF4 positively correlated tightly (r=0.94, P<0.0001) with TGF-β_1_ concentration (**Figure 4D**). Taken together, these data indicate that platelets are highly likely to be an important source of the TGF-β_1_ present at elevated concentrations in plasma in acute, subacute and chronic HIV-1 infection.

### DCs Do Not Produce TGF-β_1_, Activins A and B or BMP-2 Following *In Vitro* Exposure to HIV-1

DCs are known to be rapidly activated during acute HIV-1 (and also SIV) infection (41–43). As DCs constitute an important cellular source of innate cytokines, and are known to be capable of producing (and responding to) a number of TGF-β superfamily cytokines (17, 44), *in vitro* studies were conducted to determine whether production of TGF-β superfamily cytokines is triggered following exposure of primary pDCs or mDCs to HIV-1, TLR7/8 ligands [a surrogate for HIV-1 genomic RNA (45, 46)] and synthetic ligands for other TLRs expressed by these DC subsets. Although other leukocyte subsets, such as Treg cells, constitute an important source of TGF-β_1_ ****in HIV and SIV infections (47), these cells are not directly activated by HIV-1 and are therefore unlikely to be the main source of the TGF-β_1_ released into the circulation at the earliest timepoints after viremic HIV-1 spread begins to occur, so were not studied here.

Stimulation of pDCs with HIV-1, influenza virus (the genome of which also serves as a TLR7 ligand) or synthetic TLR7 (R837) or 9 (ODN2216) ligands elicited robust production of IFN-α, but did not trigger release of TGF-β_1_, activins A or B or BMP-2 (**Figure 5**). HIV-1 exposure also failed to elicit production of TGF-β_1_, activins A or B or BMP-2 from mDCs (**Figure 6**), although mDCs did produce significant quantities of activin A in response to stimulation with the TLR4 ligand LPS [consistent with prior reports (44, 48)], and also released some activin A following exposure to a synthetic TLR7/8 ligand (R848).

## Discussion

This study addressed perturbations in systemic levels of diverse TGF-β superfamily members (TGF-β_1,_ activins A and B and BMP-2) during the critical initial stages of HIV-1 infection and at time-points during subacute and chronic infection. Circulating concentrations of many other innate cytokines are transiently upregulated in a “storm” that begins ~7 days after first detection of viremia and continues until just after the peak virus replication, then normalize or exhibit less pronounced elevations during subacute and chronic infection. However, TGF-β superfamily cytokines were found to behave distinctly. A moderate increase in plasma TGF-β_1_ levels occurred as early as 1 to 4 days after first detection of viremia and persisted throughout the acute phase of infection, with elevations in circulating TGF-β_1_ concentrations also being present during subacute and chronic infection. By contrast, no substantial perturbations were detected in systemic levels of the other TGF-β superfamily members analyzed during acute or chronic infection, although there was a trend for an increase in circulating activin A and B concentrations during chronic infection. Notably, *in vitro* analyses revealed that HIV-1 exposure of DC subsets, which comprise an important source of cytokine production during the acute phase of HIV-1 infection, did not elicit TGF-β superfamily cytokine release, providing a putative explanation for the lack of a profound systemic upregulation of these cytokines during the innate cytokine “storm” in acute HIV-1 infection. However, the marked positive correlation between platelet activation and plasma TGF-β_1_ levels in HIV-1 infection suggests that platelets may comprise an important source of sustained TGF-β_1_ upregulation in HIV-infected individuals.

The kinetics of systemic elevation of TGF-β_1_ during HIV-1 infection defined in this study parallel those reported in pathogenic non-human primate infection models such as SIVmac infection of rhesus macaques, where a modest increase in circulating TGF-β_1_ levels is observed from as early as one day post-infection and is maintained throughout acute/subacute and into chronic infection (24), and increased transcription of genes downstream of TGF-β signaling follows similar kinetics (49). Circulating TGF-β_1_ levels in the earliest stages of acute HIV-1 infection have not previously been investigated, but our observation of elevations in plasma TGF-β_1_ at later stages infection is consistent with findings from a number of other studies (26, 27, 50–53). The modest and sustained systemic upregulation of TGF-β_1_ in HIV-1 and pathogenic SIV infection contrasts sharply with the pattern of TGF-β_1_ in non-pathogenic SIVagm infection of African green monkeys, where a robust CD4^+^ Treg cell response is activated one day after infection, with an associated pronounced increase in systemic TGF-β_1,_ but circulating TGF-β_1_ concentrations during subacute and early chronic infection are in fact reduced compared those present prior to infection (24).

The differing kinetics of TGF-β_1_ production in pathogenic and non-pathogenic immunodeficiency virus infections raise the hypothesis that like type I IFNs, TGF-β_1_ may play both beneficial and detrimental roles during infection, with early induction contributing to protection whilst sustained upregulation has pathogenic consequences. *In vitro* studies suggest that TGF-β_1_ may have complex, cell-type dependent effects on HIV-1 replication. In monocytes/macrophages it enhances HIV-1 replication (54–57). Conversely, in Langerhans cells TGF-β_1_ restricts HIV-1 infection by inducing a post-entry block to virus replication (58). Moreover, by limiting immune activation very early after infection TGF-β_1_ may putatively reduce the availability of activated CD4^+^ cells to support HIV-1 replication and spread, although this has not been systematically investigated. During the initial stages of HIV-1 infection, when Langerhans cells may be infected and virus amplification in CD4^+^ T cells is critical, TGF-β_1_ may thus have a beneficial role overall. However, the sustained production of TGF-β_1_ throughout the acute/subacute phases of SIVmac infection of rhesus macaques was found to be associated with upregulation of Smad7, an inhibitor of TGF-β signaling, raising the hypothesis that early protective effects of TGF-β_1_ may become blunted by subacute infection due to leukocytes becoming less responsive to this cytokine (59).

Although TGF-β may play a role in limiting initial virus replication, given its ability to suppress the responses of multiple leukocyte subsets (60) it may indirectly enhance virus replication by limiting adaptive responses that play a key role in HIV-1 control, including both humoral and virus-specific CD8^+^ T cell responses, and this detrimental activity may start to predominate in early chronic infection. The importance of TGF-β in regulation of CD8^+^ T cell responses during chronic viral infection has been directly demonstrated in murine models (61); moreover, in SIV-infected macaques virus-specific CD8^+^ T cell frequencies correlate negatively with those of CD4^+^ Treg cells, a prominent source of TGF-β (62). Chronic production of TGF-β may also have other detrimental effects, e.g. TGF-β mediates fibrosis of lymphoid tissue in HIV-1 infection (63). We did not observe any relationship between plasma TGF-β_1_ levels during subacute or chronic HIV-1 infection and viral load or CD4^+^ T cell count in the relatively small cohort of individuals studied here, but in larger studies systemic TGF-β_1_ has been found to be inversely correlated with CD4 count (26, 27), likewise circulating TGF-β concentrations in individuals with nonprogressive HIV-1 infection are reported to be lower than in progressors (26). These observations are consistent with TGF-β playing a pathogenic role during infection; although it cannot be excluded that TGF-β upregulation is solely a marker of ongoing immune activation and associated disease progression rather than a determinant thereof. Pathogenic and non-pathogenic immunodeficiency virus infections are distinguished by sustained upregulation of IFN-stimulated gene (ISG) signatures in blood and lymphoid tissue cells in the former but not the latter (15, 16, 64). Given the ability of TGF-β superfamily members including activins and BMPs to promote ISG expression via both IFN-dependent and -independent mechanisms in certain cell types (22, 65), it could be speculated that sustained TGF-β_1_ production during chronic infection may play a role in potentiating this pervasive, disease-associated ISG activation signature.

Notably, no substantial systemic elevation of activin A, activin B or BMP-2 was observed at any stage of HIV-1 infection, although there was a trend for higher circulating levels of activins A and B at later stages of infection. By contrast, multiple other innate cytokines are rapidly upregulated during acute HIV-1 infection and/or are more modestly elevated at later stages of infection (4,5.6,37,38). Notably, plasma activin A levels have been shown to increase in hepatitis B and C infections (66, 67), during septicemia (68), and it contributes to inflammatory cascades and acute-phase protein upregulation (69). Given the intense immune activation and acute-phase protein production observed in HIV-1 infection, a highly inflammatory state (4, 6, 36, 70), the lack of pronounced activin A upregulation is striking.

DCs are key cellular sources of innate cytokine production in acute HIV-1 infection (41). Plasmacytoid DCs are directly activated by HIV-1, which binds to and is endocytosed by these cells, enabling TLR7-mediated sensing of the viral RNA genome (45). Proinflammatory cytokine release by pDCs then triggers cytokine production by myeloid DCs that are not directly activated by HIV-1 exposure (41). We showed that pDCs did not produce activin A or other TGF-β superfamily cytokines following *in vitro* exposure to HIV or other activating stimuli that triggered potent type I IFN release from these cells; likewise, other studies similarly suggest that pDCs do not produce activin A (44, 71). This could explain the lack of systemic activin upregulation co-incident with type I IFN production as viremia first starts to increase in acute HIV-1 infection. However, mDCs are abundant producers of activin A. Unsurprisingly, HIV-1 exposure did not elicit activin release from mDCs, but activin A production was triggered by LPS, consistent with prior observations (44, 48). The trend for an increase in circulating activin A levels we observed in chronic HIV-1 infection may potentially be driven by LPS, which is known to translocate into circulation as a consequence of damage to gut-associated lymphoid tissues during the initial stages of infection (72). However, although HIV-1 does not directly stimulate mDCs, mDC release of activin A can be triggered indirectly by tumor necrosis factor (TNF) (44); furthermore macrophages (73) and neutrophils (74) can also be stimulated to release activin A in the context of inflammation. This makes the lack of detectable systemic increase in activin A during the cytokine “storm” in acute infection when pro-inflammatory cytokines including TNF are known to be upregulated (4, 75) particularly remarkable.

TGF-β is prominently produced by T cells, particularly Treg cells (76). However, these cells likely do not represent the source of the earliest elevations in TGF-β_1_, as they are not directly stimulated by HIV-1 unless they have been pre-primed to respond to viral antigens. Accordingly, in one *in vitro* study where HIV-1 antigens were shown to induce TGF-β_1_ production from CD4^+^ and CD8^+^ Tregs from HIV-infected individuals within 24 h of stimulation, the source implicated was pre-established HIV-specific Tregs (77). In another study that stimulated PBMCs from HIV-seronegative donors with HIV-1 Env proteins and found production of TGF-β_1_ within 48 h, TGF-β_1_ induction was no longer observed when analyzing CD2-enriched cells from the same donors (78). Thus, Tregs are less likely to constitute the main source of circulating TGF-β_1_ in HIV-seronegative individuals in the earliest stages of infection. Nonetheless, Tregs likely do contribute to TGF-β_1_ production at later times post-infection.

Platelets are an abundant cellular source of TGF-β_1_ that is released upon activation and degranulation (79). The very rapid increase observed in plasma TGF-β_1_ following the onset of viremia (which precedes activation of DCs and T cells), together with the strong correlation between TGF-β_1_ and PF4 levels suggest that platelets are likely to be a dominant source of the TGF-β_1_ found in the circulation during HIV-1 infection. Platelets can be artefactually activated after blood withdrawal due to the phlebotomy technique, physical agitation of samples, incorrect sample storage or improper platelet depletion during processing (80), and anticoagulants are also documented to cause some degree of platelet activation *ex vivo* (81, 82). PF4 is sometimes referred to as a marker of artefactual *ex vivo* platelet degranulation owing to its very short half-life *in vivo* (83). However, it is highly unlikely that the elevations in plasma TGF-β_1_ observed in plasma samples from HIV-infected individuals are an artefact of *ex vivo* degranulation, as baseline and post-viremic plasma donor samples, and samples from HIV-1-seronegative, subacute and chronically infected individuals were acquired and processed in a similar fashion, but time and group-dependent differences, respectively, were nonetheless observed. Furthermore, systemically elevated TGF-β_1_ in HIV-infected individuals was also documented in a prior study where a robust platelet depletion protocol was employed (26). Together these observations support the hypothesis that parallel elevations in both TGF-β_1_ and PF4 are elicited as a consequence of *in vivo* platelet activation during HIV-1 infection.

Notably, increased platelet activation in HIV-infected individuals has also been reported in other studies (84–88), and in SIV-infected macaques (89). The HIV-1 Tat protein activates platelets (90), and mice injected with Tat show a marked increase in plasma PF4 within 1 h (91, 92), suggesting that Tat may be an important trigger of platelet activation to release TGF-β_1_ prior to the activation of cellular innate responses as viremia begins during acute HIV-1 infection. Other mechanisms that may contribute to ongoing platelet activation in HIV-1 infection could include production of TNF, which activates platelets (93); and of platelet-activating factor (PAF), the bioactivity of which is increased in HIV-1 infection (94).

The demographics of the longitudinally-studied US plasma donors were unknown; but the cross-sectional plasma samples from subacute and/or chronically-infected UK donors were derived from Caucasian MSM. Important differences are observed in some aspects of the host immune response in individuals of differing sex or ethnicity. For example, pDCs of women produce more IFN-α than those of men when stimulated with HIV-1 (95), and in AHI, women generally establish a lower persisting viral load than men, but are then likely to progress to AIDS more rapidly than men with similar viral loads (96). It is thus important to consider how the demographics of the subjects studied here may have influenced the observations made. No difference has been observed in circulating levels of TGF-β_1_ in male and female subjects in prior studies (79, 97, 98), but differences have been reported in subjects of differing ethnicity, with higher circulating TGF-β_1_ observed in black compared to white ethnic groups (99). However, although the chronically-infected subjects studied here were predominantly Caucasian, plasma TGF-β_1_ levels in chronically HIV-1–infected subjects were also found to be higher than those in seronegative controls in another study where all participants were of black African ethnicity (51), and similar observations were also made in a study focusing on a relatively mixed group of Caucasian, Asian and black subjects (53). Thus, despite differences in baseline expression levels of TGF-β_1_ between ethnic groups, the perturbations in systemic TGF-β_1_ concentrations at different stages of HIV-1 infection observed in our study likely still occur in all infected individuals regardless of ethnicity.

In summary, we have demonstrated a very rapid systemic upregulation of TGF-β_1_ in acute HIV-1 infection, likely due to platelet activation, which persists through subacute and chronic infection. By contrast, we show that other members of the TGF-β superfamily are not substantially induced during acute/subacute infection, although low-level activin A/B may start to occur during chronic infection, putatively as a consequence of LPS-driven mDC stimulation of activin production. Further work is needed to define the effects of TGF-β superfamily cytokines on HIV-1 replication, but as most members of this family are not produced at high levels during HIV-1 infection, if they do mediate antiviral activity against HIV-1 they could be exploited to restrict HIV-1 replication. Moreover, if sustained TGF-β_1_ upregulation contributes to driving expression of inflammatory/IFN-ISG signatures in chronic infection, blocking the induction or activity of this cytokine could be considered to decrease excessive immune activation and associated pathology.

## Data Availability Statement

The raw data supporting the conclusions of this article will be made available by the authors on request, without undue reservation.

## Ethics Statement

All human samples used in this study were obtained with appropriate ethical approval from relevant ethics committees (Local Research Ethics Committee for Camden and Islington and National Health Service Health Research Authority South Central – Berkshire Research Ethics Committee for the samples from UK HIV-infected individuals and demographically-matched seronegative controls), and all study subjects provided written informed consent.

## Author Contributions

MD designed experiments, conducted experiments, acquired and assimilated data, and co-wrote the manuscript. AK produced HIV-1 NL4-3 from IMCs and quantified reverse transcriptase. EG performed statistical analysis of data. DP, PP, and IW provided clinical samples. HD advised on experimental design and provided feedback on the manuscript. PB conceived and directed the study, designed experiments, directed the assimilation of the data, provided reagents, and co-wrote the manuscript. All authors contributed to the article and approved the submitted version.

## Funding

This work was supported by funding from the NIH, NIAID, DAIDS (R01 AI 114266) and the Medical Research Council (MR/K012037). PB is a Jenner Institute Investigator.

## Conflict of Interest

The authors declare that the research was conducted in the absence of any commercial or financial relationships that could be construed as a potential conflict of interest.
