# Supplementary material for: Dynamics of Transforming Growth Factor (TGF)-β Superfamily Cytokine Induction During HIV-1 Infection Are Distinct From Other Innate Cytokines
Source: Front Immunol. 2020 Nov 24;11:596841. doi: 10.3389/fimmu.2020.596841 (PMC7732468; doi:10.3389/fimmu.2020.596841)
Supplement: Supplementary file 1 [file DataSheet_1.pdf]

Dickinson et al Supplementary Figure 1

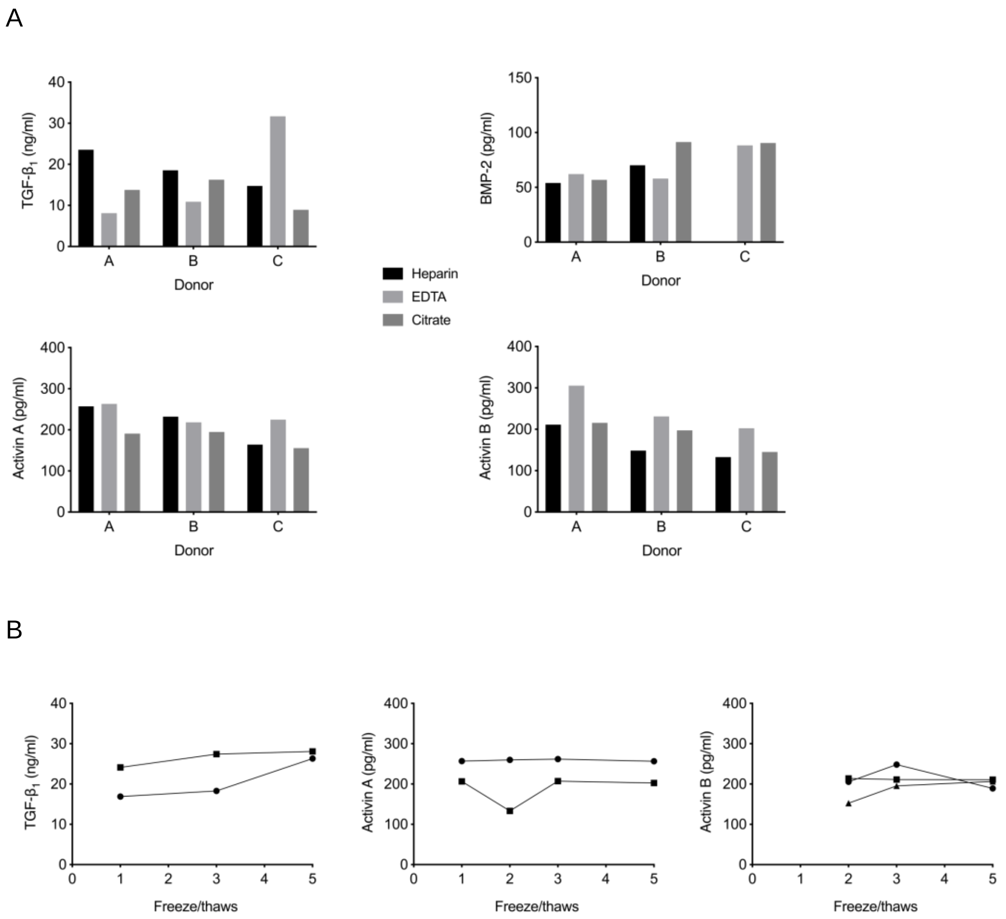

Supplementary Figure 1: Analysis of the effects of anticoagulants and freeze-thawing on TGF-β superfamily cytokine concentrations measured by ELISA or Luminex assay

A. Plasma separated from blood samples collected in heparin, EDTA or 4% sodium citrate was analyzed to determine levels of TGF-β_1 (Luminex assay), BMP-2, activin A or activin B (ELISA). Samples were assayed in technical duplicate and the results averaged. CV percentages of greater than 20% between technical replicates were rejected, resulting in the omission of one sample (BMP-2, heparin, donor C). **B.** Plasma separated from blood collected into heparin anticoagulant was spiked with recombinant TGF-β_1 (5ng/ml), activin A (100pg/ml) or activin B (100pg/ml), then serially freeze-thawed up to 5 times before measurement of those cytokines by Luminex assay or ELISA. Each unique symbol represents data from plasma from a different donor.
